# Mesenchymal Stem Cells: A Potential Therapeutic Approach for Amyotrophic Lateral Sclerosis?

**DOI:** 10.1155/2019/3675627

**Published:** 2019-03-10

**Authors:** Agnese Gugliandolo, Placido Bramanti, Emanuela Mazzon

**Affiliations:** IRCCS Centro Neurolesi “Bonino Pulejo”, 98124 Messina, Italy

## Abstract

Amyotrophic lateral sclerosis (ALS) is a neurodegenerative disease characterized by the degeneration of both upper and lower motor neurons. Patients show both motor and extra-motor symptoms. A cure is not available at this time, and the disease leads to death within 3–5 years, mainly due to respiratory failure. Stem cell therapy is arising as a new promising approach for the treatment of neurodegenerative disorders. In particular, mesenchymal stem cells (MSCs) seem the most suitable type of stem cells, thanks to their demonstrated beneficial effects in different experimental models, to the easy availability, and to the lack of ethical problems. In this review, we focused on the studies involving ALS rodent models and clinical trials in order to understand the potential beneficial effects of MSC transplantation. In different ALS rodent models, the administration of MSCs induced a delay in disease progression and at least a partial recovery of the motor function. In addition, clinical trials evidenced the feasibility and safety of MSC transplantation in ALS patients, given that no major adverse events were recorded. However, only partial improvements were shown. For this reason, more studies and trials are needed to clarify the real effectiveness of MSC-based therapy in ALS.

## 1. Introduction

Amyotrophic lateral sclerosis (ALS) is a lethal neurodegenerative disorder characterized by the selective degeneration of both upper (UMN) and lower motor neurons (LMN), causing both motor and extra-motor symptoms [1, 2].

LMNs are in the brainstem and spinal cord and transmit impulses from the UMNs to the muscles at the level of the neuromuscular synapses to innervate the skeletal muscles that control the arms and the legs. The main symptoms of ALS are muscle weakness, wasting, in particular in the limbs, cramps, twitching, and problems in speaking [3]. Specifically, UMN symptoms include weakness, speech difficulties, spasticity, and inappropriate emotionality, while LMN symptoms are represented by cramps, twitching, muscle wasting and weakness [3]. The patients can show an initial presentation with spinal-onset disease, which is the most common form characterized by limb muscle weakness, or with bulbar-onset disease, whose characteristics are dysarthria and dysphagia [2, 3]. In Europe, the ALS incidence was estimated to be 2 to 3 cases per 100,000 individuals [2].

However, other than by a progressive and asymmetric weakness and atrophy in limb, thoracic, abdominal, and bulbar muscles, ALS is associated also with extrapyramidal features, postural abnormalities, small fiber neuropathy, and mild oculomotor disturbance [1]. Even if ALS main symptoms are correlated with motor dysfunction, about 50% of patients show also cognitive and behavioural impairment [2].

Usually, ALS leads to death within 3–5 years [1, 2]. Respiratory failure is recognized as one of the main complications of ALS and one of the main causes leading to death [4, 5]. The appearance of respiratory failure is caused by the impairment of the respiratory musculature, and it is influenced by the concomitant presence of other respiratory pathologies [4, 5]. The loss of function of the phrenic nerve induces diaphragm weakness, leading as a consequence to dyspnea, orthopnea, and hypoventilation [4]. Unfortunately, respiratory symptoms are not easy to recognize and alterations of blood gas analysis become evident only in a late stage of the disease or when an acute episode of respiratory failure happens [5]. Noninvasive ventilation, considered a standard treatment for respiratory support for ALS patients, was demonstrated to improve the quality of life but also to increase the survival of patients [4, 5].

The cause of ALS is not clear. The familial forms of ALS are reported only in 5–10% of cases; on the contrary, the majority of ALS cases are sporadic. Regarding the familiar form, the genes mainly involved are SOD1, TARDBP, FUS, OPTN, VCP, UBQLN2, C9ORF72, and PFN1 [6]. In particular, it is known that about two-thirds of familial cases are caused by the genes C9ORF72, SOD1, TARDBP, and FUS [7].

Furthermore, epidemiological and experimental studies evidenced also the influence of environmental and lifestyle factors in the ALS pathogenesis, such as dietary factors, pesticide or heavy metal exposure, smoking, alcohol, viral and fungal infections, physical exercise, and electromagnetic radiations [8, 9]. In particular, some workers may present a higher risk of developing ALS, such as athletes, carpenters, construction, electrical, and farm workers, and others, due to the occupational prolonged exposition to chemicals, pesticides, metals, or electromagnetic radiations [9].

Even if ALS pathogenesis is not fully clear, some of the pathogenic processes that are involved include excitotoxicity, neuroinflammation, mitochondrial dysfunction, and protein misfolding [1]. Notably, even if ALS is characterized by the death of motor neurons, a wide variety of studies have shown that nonneuronal cells, such as astrocytes and microglia, may contribute to the injury and death of motor neurons, through the so called non-cell autonomous processes [10].

A curative treatment for ALS, able to block disease progression, has not been developed yet. Nowadays, only 2 drugs are approved by the Food and Drug Administration (FDA) for ALS treatment: riluzole and edaravone [11]. Riluzole is a glutamate antagonist whose mechanism of action is not fully clear, but it is known to inhibit glutamate release, inactivate voltage-dependent sodium channels, and interfere with intracellular events after the activation of excitatory amino acid receptors. Interestingly, it is able to extend the survival of ALS patients [11, 12]. Edaravone was recently approved by FDA and acts as an antioxidant. It is thought to be a free radical scavenger and may exert its action reducing oxidative stress in motor neurons and glial cells [11, 13]. In the context of developing new therapeutic strategies, stem cell therapy is reaching promising results in the treatment of different neurodegenerative disorders. The assumption of this approach is that stem cells, which are able to differentiate into neuronal cells, may replace the degenerated neurons. Moreover, stem cells are able to release different growth factors that may create a microenvironment that promotes neuroprotection. In this regard, different types of stem cells may be used, with mesenchymal stem cells (MSCs) representing a most suitable type. Indeed, MSCs are multipotent, self-renewal stem cells, characterized by an easy accessibility and expandability *in vitro*, and all these properties make MSCs a good source of stem cells for cell therapy and regenerative medicine. In addition, being adult stem cells, MSCs do not face the ethical concerns linked to the use of embryonic stem cells.

## 2. Mesenchymal Stem Cell Characteristics

MSCs are multipotent stem cells that are able to transdifferentiate into various lineages [14, 15]. The minimal criteria proposed by the “Mesenchymal and Tissue Stem Cell Committee of the International Society for Cellular Therapy” to define MSCs are the following:

- In standard culture conditions, they are plastic-adherent cells
- They express CD105, CD73, and CD90, but no CD45, CD34, CD14 or CD11b, CD79alpha or CD19, and HLA-DR surface molecules
- They are able to differentiate into osteoblasts, adipocytes, and chondroblasts *in vitro* [16]

**Figure 1:**
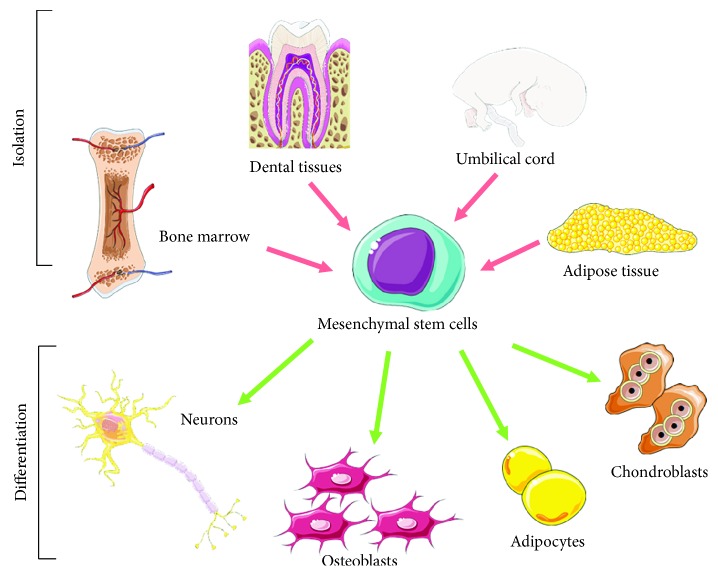
Isolation and differentiation potential of mesenchymal stem cells (MSCs). MSCs may be isolated from various sources, such as bone marrow, adipose tissue, umbilical cord, and dental tissues. Furthermore, MSCs may differentiate toward different lineages, such as neuronal cells, osteoblasts, adipocytes, and chondrobasts. The image was created using the image bank of Servier Medical Art (available at http://smart.servier.com/), licensed under a Creative Commons Attribution 3.0 Unported License (https://creativecommons.org/licenses/by/3.0/).

MSCs may be isolated from various sources, such as bone marrow [17], umbilical cord [18], dental tissues [19], adipose tissue [20] (Figure 1), and others [21]. Bone marrow-derived MSCs (BMMSCs) were the first MSCs to be discovered, and for this reason BMMSCs are the most characterized and studied. However, MSCs derived from other sources may have different advantages. Adipose tissue permits to obtain large amounts of MSCs with an easier isolation procedure, and even if adipose tissue-derived MSCs (ADMSCs) share different biological features with BMMSCs, some differences exist [22, 23]. Dental-derived MSCs origin from the neural crest, and for this reason they may represent a good MSC source for neurological application [24, 25]. On this base, it would be opportune to choose the most appropriate source of MSCs depending on the application.

MSCs can differentiate into all the three lineages mesoderm, endoderm, and ectoderm, including toward neuronal cells [15] (Figure 1), thanks to the addition of specific substances. In particular, different molecules and growth factors can be used to induce the neural differentiation of MSCs [26]. Notably, MSCs derived from different tissues were reported to be able to differentiate toward motor neurons through different protocols, as demonstrated by the expression of neuronal markers [27–30]. Human umbilical cord blood-derived MSCs differentiated into motor neurons after the exposure to retinoic acid (RA), sonic hedgehog (Shh), and brain-derived neurotrophic factor (BDNF) using a three-step *in vitro* procedure. Cells showed a bipolar morphology and expressed markers associated to motor neurons [28]. BMMSCs can be differentiated in functional motor neuron-like cells using *β*-mercaptoethanol, RA, Shh, and nerve growth factor (NGF) [29]. Chorion-derived MSCs can differentiate into motor neuron-like cells in the presence of RA and Shh [30]. Furthermore, functional motor neuron-like cells may be obtained using genetically engineered MSCs, in order to express transcription factors associated to motor neurons, and after being treated with a specific inductive medium [31].

However, their differentiation capacity is not the only property that explains their beneficial action in the different pathological contexts. Indeed, it is known that MSC activity is mediated at least in part in a paracrine manner, through the release of different factors and biomolecules that represent the MSC secretome. These secreted factors included soluble proteins, such as growth factors and cytokines, free nucleic acids, lipids, and extracellular vesicles. The biomolecules included in secretome derivatives, such as conditioned medium (CM) or exosomes, may promote tissue repair and mediated immunomodulation, anti-inflammatory, and antiapoptotic activities [32]. The anti-inflammatory and neuroprotective actions of the secretome may be mediated at least in part through the release of anti-inflammatory cytokines. In particular, transforming growth factor *β* (TGF-*β*), interleukin (IL) 10, and other anti-inflammatory cytokines may be responsible for the anti-inflammatory action, while neurotrophic factors may mediate neuroprotection [32]. Other than soluble factors, MSCs release exosomes, which are membrane-enclosed vesicles, surrounded by a phospholipid layer that contained proteins, lipids, and coding and noncoding RNAs. Interestingly, the administration of isolated MSC-derived exosomes has been demonstrated to exert beneficial actions in different models and in regenerative medicine [33].

Another important feature of MSCs, particularly useful in the clinical context, is their capacity to migrate to the site of injury after their administration, a property called “homing” mediated by chemokines [34, 35].

Thanks to all their beneficial properties, MSCs have already shown promising results in the treatment of different neurodegenerative diseases, such as Parkinson's disease [36, 37], Alzheimer's disease [38], and multiple sclerosis [39].

## 3. MSC Application in ALS Rodent Models

Different studies evaluated the efficacy of MSC administration in ALS rodent models. The most used ALS animal models are the SOD1 transgenic ones, developed after the discovery that the SOD1 gene is responsible for familial forms of ALS. In particular, the transgenic SOD1^G93A^ animal model that carries the substitution of the amino acid glycine with alanine at residue 93 is widespread [40, 41]. SOD1 transgenic models show different features of the ALS pathology, such as the progressive loss of motor neurons, axonal and mitochondrial dysfunction, progressive motor paralysis, muscle atrophy, and reduced lifespan [40–42].

MSC intravenous injection in irradiated SOD1 mice before clinical manifestation was demonstrated to be able to delay the ALS onset and to increase lifespan. In addition, the motor neuron loss in mice that received MSCs was slower and less severe compared to untreated animals, leading to a delay in motor function loss in the MSC-treated group. Interestingly, MSCs, that survived more than 20 weeks, were able to migrate into the brain and spinal cord, where some of them expressed neuroglial differentiation markers. However, MSCs were detected also in peripheral tissues, including the lung, liver, and spleen [43]. In line with this work, Vercelli and coworkers [44] also transplanted BMMSCs at the level of the lumbar spinal cord in asymptomatic SOD1 mice and found that the cells were able to survive in the spinal cord for long periods, at least 10 weeks after transplantation. MSCs transplanted into female mice, characterized by a slower disease progression, reduced microglial activation and astrogliosis and loss of motor neurons. Moreover, motor function improved in transplanted males [44]. However, less than 1% of transplanted BMMSCs were glial fibrillary acidic protein- (GFAP-) or microtubule-associated protein 2- (MAP2-) positive. For this reason, it is possible to speculate that MSC transplantation exerts a positive action increasing neuronal survival and preventing neuroinflammation instead of replacing lost neurons [44].

Interestingly, while previous reports evidenced a therapeutic role of MSC transplantation in SOD1^G93A^ mice when administered before disease onset, Uccelli et al. [45] highlighted that MSCs were able to exert a beneficial effect also when intravenously injected after the occurrence of clinical symptoms. Indeed, MSC administration in symptomatic SOD1 mice improved animal survival, but also the clinical outcome and the pathological scores, delaying disease progression and the loss of motor function. However, only a small number of MSCs migrated into the central nervous system (CNS) of the animals, suggesting that their action did not depend on long-term engraftment [45]. From a molecular point of view, a reduction of ubiquitin agglomerates was evidenced in the spinal cord of MSC-treated SOD1^G93A^ mice, with no changes in the number of cells positive for choline acetyltransferase, an enzyme specific for motor neurons, indicating no changes in the number of motor neurons after MSC transplantation. Moreover, MSC injection reduced the activation of astrocytes and microglia in the spinal cord, with the consequent decrease in the expression for the proinflammatory cytokines IL-1*β* and tumor necrosis factor *α* (TNF-*α*). MSCs modulated also oxidative stress, as demonstrated by the reduction in the increased activity of the glutathione S-transferase (GST) antioxidant enzyme and by the reversion of metallothionein upregulation. MSC treatment also reduced the excessive release of glutamate, which is upregulated in SOD1^G93A^ mice [45]. Boucherie et al., using a different route of administration, which is the injection into the cerebrospinal fluid, evidenced that MSC transplantation in symptomatic SOD1 rats was able to exert different positive actions. In particular, MSCs injected into the cerebrospinal fluid were able to infiltrate the nervous parenchyma, migrating into the ventral gray matter, where there are the degenerating motor neurons. Astrogliosis was not modified, but MSCs differentiated into astrocytes at the site of degeneration, reducing motor neuron loss in the lumbar spinal cord. On the contrary, the differentiation of MSCs into mature neurons was not observed. All these effects led to a preservation of motor function, associated with a slowing down of disease progression, given that paralysis appeared later, and to an extension of the life expectancy of SOD1^G93A^ rats. Their results suggested that the positive action of MSCs was mainly observed in the early stage of motor neuron death. In parallel to neuroprotection, a decreased inflammation was observed, as evidenced by the lower proliferation of microglia and by the decreased expression of cyclooxygenase-2 (COX-2) and NADPH oxidase 2 (NOX-2) [46].

Decreased inflammation was also reported by Boido et al. who administrated human MSCs in the cisterna lumbalis in SOD1^G93A^ mice, at the earliest onset of symptoms, and animals were killed 2 weeks after transplantation. Motor neuron death and motor function impairment were delayed, and astrogliosis was reduced. Moreover, MSC administration prevented the decrease in IL-10 and vascular endothelial growth factor (VEGF) expression and increased the expression of the anti-inflammatory cytokine IL-13 in the spinal cord [47].

Zhang et al. found that multiple intrathecal administration of human marrow stromal cells through cerebrospinal fluid increased the lifespan, protected motor neurons, and improved motor performance in SOD1 mice, even if a low number of cells was detected in the spinal cord [48]. The same group tried to explain how MSCs can exert their neuroprotective action, and they focused their attention on the anti-inflammatory role of MSCs. They found that multiple intrathecally transplanted MSCs at 8, 10, and 12 weeks of age delayed disease onset, prolonged lifespan, improved motor function, and preserved motor neurons. In addition, MSCs inhibited inflammatory response in SOD1 mice, as demonstrated by the reduction in microglial activation, TNF-*α* secretion, and inducible nitric oxide synthase (iNOS) protein levels. Moreover, through *in vitro* experiments they showed that the inhibitory action exerted by MSCs on microglial activation was due to the release of diffusible molecules [49].

Forostyak and coworkers [50] administered MSCs intraspinally and intravenously to SOD1 rats at the disease onset. 4 weeks after transplantation, an improvement in motor activity was evident in rats receiving the MSCs, associated with a higher survival and a significant elevated number of motor neurons at the thoracic and lumbar levels of the spinal cords compared with sham animals. MSCs survived in the spinal cord of transplanted animals until the end stage of the disease and migrated both rostrally and caudally from the injection site. Motor neurons were larger, and TUNEL staining was lower in the somas of apoptotic motor neurons at the thoracic level in MSC-administered animals, suggesting that implanted MSCs exerted a beneficial effect on the survival of motor neurons [50].

Kim et al. [51] evaluated if MSC effects were dose-dependent. With this aim, they administered intrathecally human BMMSCs obtained from an ALS patient in SOD1 mice. Three different doses were tested: 1 × 10^4^, 2 × 10^5^, and 1 × 10^6^. Even if there were no significant differences in the symptom onset time, they evidenced that the highest dose (1 × 10^6^ cells) was able to prolong the survival and to delay the decline of motor function, associated with a higher average number of motor neurons compared to untreated animals and to those receiving the lowest MSC dose. The group treated with 2 × 10^5^ showed only a significant difference in survival time and a decreased motor neuron loss compared to control animals. MSCs transplanted into the cisterna magna were observed in the ventricular system and in the subarachnoid space, while some cells migrated into the brain and spinal cord. These data suggested that intrathecal injection may be a good route for MSC therapy and indicated a dose-dependent effect, underlining the importance to administer an adapt number of MSCs to obtain a successful therapy [51].

Forostyak et al. studied the changes in ventral horn perineuronal nets (PNNs) of SOD1 rats during the normal disease progression and the effects of the intrathecal administration of human BMMSCs after symptom appearance [52]. The PNNs constitute a layer of condensed pericellular matrix that aggregates and wraps around the soma and proximal dendrites of some neurons, composed of hyaluronan, chondroitin sulphate proteoglycans (CSPGs), link proteins, and tenascin-R [53]. MSCs improved disease progression and motor performance and increased survival. Moreover, the injection of MSCs rescued the number of motor neurons. They were the first to report that SOD1 rats have an abnormal disorganized PNN structure around the spinal motor neurons, showing different protein and gene expression profiles of some CSPGs. However, the administration of MSCs preserved the PNN structure and modified the expression of CSPGs, indicating the reactivation of CNS plasticity, associated with a better survival of motor neurons [52].

All previous studies evaluated the effects of MSC transplantation using BMMSCs. However, MSCs derived from other sources were also tested in ALS rodent models obtaining promising results. ADMSCs, intravenously administered to SOD1 mice at the clinical onset, delayed motor function deterioration as demonstrated by clinical and neurophysiological evaluations. However, this study evidenced no difference in lifespan between treated and untreated groups. After the systemic injection, ADMSCs were able to migrate and survive into the damaged CNS but also in spleen and muscle tissues. Cells were found in the white and gray matter of the spinal cord, and their number persisted until day 135. However, ADMSCs did not differentiate toward neuronal or glial lineages and no damage, including tumor formation, was detected in the spinal cord or in muscles after implantation. Neuropathological examination of ADMSC-treated SOD1-mutant mice at day 100, the time of their best motor performance, evidenced the presence of a higher number of lumbar motor neurons. In particular, evaluating the factors that may prolong neuronal survival in spinal cord, they found the significant upregulation in the levels of glial-derived neurotrophic factor (GDNF) and basic fibroblast growth factor (bFGF) after ADMSC administration. Taking into consideration that ADMSCs produced bFGF but not GDNF in vitro, these results indicated that ADMSCs may induce neuroprotection directly, but also modulating the secretome of local glial cells toward a neuroprotective phenotype, enhancing ADMSC effects. Such neuroprotection resulted in a stronger and prolonged effect on motor performance [54].

In another study, human ADMSCs were transplanted in ALS mice intravenously or intracerebroventricularly before the symptom onset. The transplantation of ADMSCs via the intracerebroventricular route induced better results, showing a delay in disease onset and an increase in the lifespan of ALS mice. In intracerebroventricular administered mice, ADMSCs were identified in the lumbar spinal cords, but a small number of them expressed cell type-specific markers for neurons or astrocytes, indicating that only a small percentage of cells, about 1%, differentiated. Also in the spinal cord of the intravenous transplanted group, some ADMSCs were identified, but with a very low positivity for neuronal markers. Moreover, ADMSCs were found to secrete high levels of neurotrophic factors such as NGF, brain-derived neurotrophic factor (BDNF), insulin-like growth factor 1 (IGF-1), and VEGF. These factors reduced apoptotic cell death as confirmed in cultured primary spinal cord cells and in the ALS spinal cord of the group of mice that received MSCs intracerebroventricularly. Altogether, these results indicated that the transplantation of ADMSCs in ALS mice exerted neuroprotective effects through the production of cytokines and growth factors, delaying disease progression and prolonging the survival of ALS mice [55].

In order to evaluate MSC distribution after administration, umbilical cord MSCs (UC-MSCs) were labelled with fluorescent poly(methyl methacrylate) nanoparticles (PMMA-NPs) and their distribution was evaluated at different time points. In particular, UC-MSCs were injected intravenously or administered through lateral ventricles and transplanted in healthy or early symptomatic SOD1^G93A^ mice. After intravenous administration, cells were sequestered by the lungs and the liver. A week after UC-MSC injection, the pattern of signal distribution was similar but a lower fluorescence intensity was detected. A further strong reduction in fluorescence was observed three weeks after cell administration. Moreover, no difference in the distribution was found among healthy and SOD1^G93A^ mice. On the contrary, UC-MSCs transplanted in lateral ventricles were found in the choroid plexus for the whole study but a decrease in number was observed. Interestingly, few UC-MSCs were observed in the spinal cord of SOD1^G93A^ mice exclusively. These results indicated that the transplantation in brain ventricles permits a longer permanence of MSCs near the injured tissues [56].

The same group evidenced that UC-MSCs administered intracerebroventricularly for 3 times were able to exert a protection of motor neurons in the lumbar spinal cord of SOD1^G93A^ mice, even if it was partial, reducing motor neuron loss. UC-MSCs reduced neuroinflammation in the spinal cord, promoting the change from a proinflammatory to an anti-inflammatory microenvironment, as demonstrated by the reduction in activated microglia and in gene expression of proinflammatory cytokines, while the expression of anti-inflammatory interleukins and IGF-1 increased. The beneficial effects may be mediated by the activation of the p-Akt survival pathway in motor neurons and astrocytes. However, UC-MSC administration did not prevent muscle denervation and disease progression in SOD1^G93A^ mice [57].

Sun and coworkers evaluated the effects of multiple administration of human amniotic MSCs (AMSCs) in SOD1^G93A^ mice. Cells were administered at the onset, progression, and symptomatic stages of the disease. AMSCs were observed in the spinal cord at the end stage of the disease, but they did not differentiate either toward neurons or toward glial cells as demonstrated by the negativity for *β*-tubulin III and GFAP. Moreover, AMSCs were found in the lung. They demonstrated that multiple AMSC injections significantly delayed disease progression, prolonged lifespan, improved motor function, prevented motor neuron death in the lumbar spinal cord, and prevented weight loss. In addition, AMSC multiple administration reduced microglial activation and reactivated astrogliosis in the spinal cord, showing to be able to reduce neuroinflammation [58].

In order to improve MSC performance, Suzuki et al. performed an intramuscular transplantation of MSC engineered to secrete glial cell line-derived neurotrophic factor (GDNF) bilaterally into three muscle groups [59]. Before MSC transplantation, a focal injury to the muscles was performed in order to improve MSC integration. Maybe the injury may create a better environment thanks to the secretion of cytokines and growth factors allowing cell survival and integration. GDNF was shown to be able to protect motor neurons in a SOD1 transgenic model [60]. The engineered MSCs were able to survive in the muscles, where they released GDNF, increasing the neuromuscular connections and the number of motor neuron cell bodies in the spinal cord at the middle stages of the disease. The transplantation of MSCs releasing GDNF also delayed disease progression and increased animal survival [59].

Chan-Il and coworkers examined the effect of neural induction on the therapeutic potential of MSCs. Human BMMSCs were neurally induced by stably transducing with a retroviral vector encoding neurogenin 1 (Ngn1) and transplanted in ALS mice. A group of animals was transplanted with untreated MSCs in order to compare the efficacy of unprocessed and neutrally induced MSCs (MSCs-Ngn1). Interestingly, they noticed that MSCs-Ngn1 were detected in the CNS in a bigger number compared to animals injected with unprocessed MSCs. The stronger tropism of MSCs-Ngn1 for the CNS of ALS mice may be due to the interaction between the MSCs chemoattractant MCP-1, expressed at higher levels in ALS mice, and its receptor CCR2. Indeed, neural induction of MSCs with Ngn1 upregulated the expression levels of CCR2, compared to untreated MSCs. MSCs-Ngn1 prevented motor neuron loss, delayed disease onset, and prolonged survival if transplanted during the preonset stage, while unprocessed MSCs failed. After, they investigated the influence of transplantation timing on disease, transplanting cells near the onset stage. In this case, transplantation of MSCs-Ngn1 increased the average lifespan. Results indicated that neural induction with Ngn1 improved the therapeutic effects of MSCs in delaying disease progression. If transplanted near the onset, a single treatment with MSCs-Ngn1 was sufficient to improve motor functions during the symptomatic stage, while unprocessed MSCs needed multiple transplantation to obtain a similar level of motor improvement [61].

Interestingly, it was suggested that magnetic resonance imaging- (MRI-) derived biomarkers can be used to identify differences between stem cell-treated and untreated SOD1^G93A^ mice [62].

A summary of the studies involving MSC administration in *in vivo* ALS rodent models is shown in Table 1.

## 4. Is Autologous MSC Therapy Possible in ALS Patients?

Thanks to the promising results obtained in ALS rodent models, different clinical trials evaluated the safety and the therapeutic effects of MSC administration in ALS patients. However, different studies that compared the characteristics of MSCs obtained from ALS patients with those obtained from healthy donors raised doubts about the feasibility of autologous MSC therapy in ALS patients. Indeed, a vantage of MSC stem cell therapy is the possibility of an autologous transplantation, where MSCs may be obtained from the same patient. However, it was necessary to evaluate if MSCs derived from ALS patients may be useful for stem cell therapy and able to exert the same effects of MSCs obtained from healthy donors. For this reason, a work evaluated the BMMSCs isolated from sporadic ALS patients and compared them with MSCs isolated from healthy donors. The results indicated that the extensive *in vitro* expansion of patient MSCs did not cause functional modifications of the cells. Indeed, no differences in morphology, differentiation potential, chromosomal alterations, and immunophenotype analysis were observed in the MSCs isolated from donors or patients. The cellular expansion potential of MSCs from patients was lower compared to donors, but the difference was not statistically significant and may be explained by the different median ages of the subjects belonging to the 2 groups [63]. Another study confirmed the usefulness of autologous cells for ALS cell therapy. Indeed, there were no significant differences between MSCs from ALS patients and healthy subjects in the expression of phenotypic markers, growth capacity, metabolic activity, osteogenic differentiation potential, and immunoregulatory properties, indicating that MSCs from ALS patients presented comparable features and functional properties compared with MSCs obtained from healthy donors. However, MSCs isolated from patients showed a higher differentiation potential into adipocytes, a higher gene expression for IL-6, but a lower production of hepatocyte growth factor compared to MSCs obtained from controls [64]. MSCs from ALS patients presented similar immunomodulatory effects compared with those obtained from healthy subjects, but respond differently to stimulation with proinflammatory cytokines [65]. Choi et al. showed that MSCs from ALS patients at earlier passages seem more appropriate for stem cell therapy in ALS patients thanks to their stability and stronger anti-inflammatory and neuroprotective actions. Indeed, the levels of different cytokines and growth factors were reduced with increasing passages [66].

However, some studies found that MSCs derived from ALS patients showed reduced properties compared to healthy MSCs. MSCs obtained from the ALS rat model SOD1^G93A^ showed an *in vitro* impairment in their neuroprotective ability [67]. Cho and coworkers found that MSCs from ALS patients showed a reduced expression of Oct-4 and Nanog and of the trophic factors ANG, FGF-2, HGF, IGF-1, PIGF, SDF-1*α*, TGF-*β*, and VEGF, but not BDNF or ECGF. Also the migration capacity was reduced [68]. In line with the previous work, Koh and coworkers suggested that pluripotency and the secretion of trophic factors of the BMMSCs derived from ALS patients were reduced in proportion to a poorer prognosis, suggesting that allogeneic BMMSCs from healthy donors may be a better option for MSC therapy in ALS patients [69].

## 5. MSC Administration in ALS Patients

Several clinical trials, approved and registered on www.clinicaltrials.gov, evaluated the administration of MSCs in ALS patients, but only for some of them the results are available and published (Table 2). In particular, the MSCs used were BMMSCs, ADMSCs, or allogeneic Wharton's jelly-derived MSCs and also different routes of administration were used in order to evaluate their safety and possible applications.

The phase I clinical trials NCT01759797 and NCT01771640 confirmed the safety and feasibility of intravenous and intrathecal injections of BMMSCs in ALS patients. Indeed, neither severe adverse events nor abnormal findings in the brain and spinal MRI scans during the 12-month follow-up after the MSC transplantation were reported. However, the ASL Functional Rating Scale (ALS-FRS) score and forced vital capacity (FVC) percentage were significantly reduced in patients belonging to both groups, indicating the progression of the disease [70]. The recent open-label clinical trial NCT01363401 determined the safety of two repeated intrathecal injections of autologous BMMSCs in ALS patients. Indeed, only transient and mild adverse events were observed, such as pyrexia, pain, and headache, but no serious adverse reactions were recorded during the 12-month follow-up period [71]. In order to evaluate disease progression, the revised ALS Functional Rating Scale (ALSFRS-R) score was determined, which measured the clinical impact of disease severity and was the most used clinical scale in clinical trials [72]. The decline in the ALSFRS-R score was not accelerated during the 6-month follow-up period, and the ALSFRS-R scores remained stable for 6 months after the initial injection of MSCs. Moreover, after intrathecal MSC injections, the cerebrospinal fluid (CFS) levels of IL-10, TGF-*β*1, TGF-*β*2, TGF-*β*3, and IL-6 increased compared with the baseline, while monocyte chemoattractant protein-1 (MCP-1), which exacerbates the motor neuron injury in ALS, was reduced and may indicate a beneficial effect on immune response in ALS patients [71]. The phase 2 randomized controlled trial NCT01363401 showed that repeated intrathecal injections of BMMSCs are safe and may exert a clinical benefit lasting at least 6 months in ALS patients. ALS patients were randomly divided into the control group, which received riluzole, and the group that other than riluzole was administered with 2 BMMSC injections. The study evidenced the lack of serious treatment-related adverse events. The mean changes in ALSFRS-R scores from baseline to 4 and 6 months after transplantation were reduced in the MSC group compared with the control group. Moreover, the MSC-administered group showed a decrease in proinflammatory cytokines and an increase in anti-inflammatory ones in the CSF. In addition, in good responders, TGF-*β*1 showed an inverse correlation with MCP-1. No significant difference was observed in the long-term survival among the 2 groups of patients. Then, the author suggested that a possible mechanism of action is that BMMSCs mediated the switching from pro- to anti-inflammatory conditions [73].

27 ALS patients were enrolled in the phase I dose-escalation safety trial NCT01609283. In this trial, 5 different dosages (ranging from 1 × 10^7^ to two doses of 1 × 10^8^ cells) of ADMSCs were administered intrathecally. The most common adverse events were temporary low back and radicular leg pain associated with the highest dose. These clinical results were associated with elevated CSF protein and nucleated cells with MRI of thickened lumbosacral nerve roots. No tumor formation was evidenced from autopsies of 4 patients. These results indicated that the intrathecal treatment with ADMSCs appeared safe. However, ALSFRS-R questionnaires showed the continued progression of disease in all treated patients. Notably, some patients reported specific mild temporary subjective clinical improvements [74].

Given that the preclinical studies have shown that neurotrophic growth factors improve the survival of motor neurons in ALS, MSCs were induced to secrete neurotrophic factors and intrathecally and/or intramuscular implanted in ALS patients in the phase 1/2 and 2a clinical trials NCT01051882 and NCT01777646. The treatment was found to be safe and well tolerated. The majority of adverse effects were mild and reversible, and not including serious adverse events. Among the most recorded adverse events, they reported headache, fever, vomiting, leg and back pain, and neck stiffness. They found no significant changes in laboratory parameters, such as blood counts, biochemistry, renal, hepatic, and thyroid functions. MRI did not show significant pathology at the site of injection in any patient [75]. The rate of progression of the FVC and of the ALSFRS-R score in the intrathecal or intrathecal and intramuscular-treated patients was reduced during the 6 months after transplantation of MSCs induced to secrete neurotrophic factors compared to the pretreatment period. 87% were classified as responders with ALSFRS-R and FVC, showing at least 25% improvement at 6 months after treatment in the slope of progression [75]. Interestingly, the trial NCT02881476 evidenced the safety of the intrathecal injection of allogeneic Wharton's jelly-derived MSCs in ALS patients [76].

Another trial (NCT02881489) showed that the intrathecal administration of BMMSCs was safe and exerted clinical benefits in a group of patients [77].

However, several studies were published regarding the administration of MSCs in ALS patients (Table 2), which may help to deepen the knowledge about the safety of MSC administration in patients. Mazzini et al. evaluated the feasibility and safety of the intraspinal cord implantation of autologous BMMSCs in ALS patients. None of the patients presented major adverse events, but only minor adverse events were recorded that were reversible. In addition, no modifications of the spinal cord volume or other signs of abnormal cell proliferation were observed [78, 79]. Among the minor adverse reactions, intercostal pain irradiation and leg sensory dysesthesia were recorded, but no severe adverse events [79]. Three months after MSC implantation, a trend towards a slowing down of the linear decline in muscular strength was observed in 4 patients in the proximal muscle groups of the lower limbs. Moreover, in 2 patients a slight increase in strength was evidenced in the same muscle groups [78]. A significant slowing down of the linear decline of the FVC and the ALS-FRS was observed in some patients after MSC transplantation [79]. No significant acute or late side effects were observed, and no modification of the spinal cord volume or abnormal cell proliferation was evidenced after a long-term follow-up. Moreover, 4 patients showed a significant slowing down of the decline of the FVC and of the ALS-FRS score, indicating that MSCs may be used for stem cell therapy in ALS [80].

A phase 1 clinical study confirmed that MSC transplantation into the spinal cord of ALS patients is safe and thus MSCs may be used in the development of ALS cell-based therapy. In particular, autologous BMMSCs, after *in vitro* expansion, were suspended in the autologous CSF and transplanted into the spinal cord. The study evidenced the lack of immediate or delayed toxicity related to MSC transplant and no serious adverse events, including tumor formation. Furthermore, given that patients received a different amount of cells, the authors evidenced that there was no correlation between the number of cells and the incidence and severity of the side effects or the outcome [81].

The safety of MSC transplantation into the CNS was evidenced during a long-term safety study. No structural changes were evidenced by MRI and no deterioration in the psychosocial status. However, no clear clinical benefits were observed [82].

A phase 1/2 open-safety clinical trial evaluated the safety and immunological effects of intrathecal and intravenous administration of autologous MSCs in patients with ALS. Fever and headache were reported mainly as adverse events, but no major adverse effects were reported in any of the patients during a follow-up of up to 25 months. The mean ALS-FRS score remained stable during the first 6 months of observation [83].

An open-label clinical study was carried out in order to identify markers of MSCs that could be used as potential predictors of response to autologous MSC therapy in ALS patients. Patients received autologous MSCs via intrathecal injection in two monthly doses, and after a 6-month follow-up, the patients were divided in responders and nonresponders based on their scores on the ALSFRS-R. In particular, as responders were classified those patients whose ALSFRS-R increased or declined more slowly during the treatment and the follow-up period. On the contrary, in nonresponders, ALSFRS-R declined at the same rate as in the expected curve or even faster. Different biological markers were measured in the MSC cultures, and their levels were compared between the responders and nonresponders. In particular, the levels of VEGF, angiogenin (ANG), and TGF-*β* in the MSC culture medium were significantly higher in responders than in nonresponders. To confirm the markers' predictive ability, MSCs isolated from one patient from each group were transplanted into the cisterna magna of SOD1^G93A^ transgenic mice. In the mouse model, the mice that received MSCs from the responder had a significantly slower onset of symptoms, a slower decline in motor function, and a significantly longer lifespan. This data suggested that VEGF, ANG, and TGF-*β* levels in MSCs may be used as potential biological markers to predict the efficacy of autologous MSC therapy and to select the patients that may have vantages from MSC administration [84].

A recent prospective, nonrandomized, open-label phase I/IIa clinical trial evaluated the safety and efficacy of autologous BMMSCs in ALS treatment. After intrathecal BMMSC application, about one third of the patients presented a mild-moderate headache, similar to those observed after a standard lumbar puncture. No serious adverse events were recorded. They observed a reduction in the ALS-FRS decline at 3 months after transplantation that, in some cases, lasts for 6 months. In the majority of the patients, FVC values remained stable or above 70% for 9 months. Values of weakness scales (WS), which evaluated muscle strength on the lower and upper extremities, were stable in 75% of patients 3 months after MSC administration. These results demonstrated that the intrathecal application of BMMSCs in ALS patients is a safe procedure and which may be useful to slow down ALS progression [85].

Another study evaluated the transplantation of bone marrow stromal cell-derived neural stem cells in spinal cord and patients were followed for a year. The procedure was shown to be safe; indeed, no patients had mortality due to operation or severe morbidity. One patient presented a temporary deterioration at the lower extremities after the implantation that improved after some weeks. The 7 patients showed a stable course after 4 months, while 5 patients were stable for 8 months after transplantation but showed a deterioration after. The study showed that intraspinal injection of bone marrow-derived neural stem cells appears to be safe [86].

Also, the combined administration of normal MSCs and MSCs committed to neuronal differentiation was revealed to be able to slow down the disease progression in 10 patients in comparison with the control group, receiving a standard symptomatic therapy, after a 12-month follow-up [87].

However, larger studies involving a bigger cohort of patients are needed to obtain meaningful results and to really understand the therapeutic potential of MSC-based stem cell therapy.

Interestingly, it was reported that MSCs may have a role in the immunomodulation of the inflammatory responses when MSC therapy is directed to ALS patients. Indeed, MSCs may exert immunomodulatory effects in ALS peripheral blood mononuclear cells (PBMCs) through the induction of regulatory T lymphocytes (Treg), increasing the levels of anti-inflammatory cytokines [88].

## 6. Future Perspectives

Nowadays, it is known that some of the beneficial properties linked to MSC use depend on the release of growth factors and soluble molecules. Moreover, some studies reported that MSCs did not survive for a long time after administration [89]. For these reasons, the research is looking for cell-free treatments based on MSC derivatives, such as MSC CM or exosomes. In particular, different studies demonstrated *in vitro* the neuroprotective effects exerted by CM on motor neuron cell lines and in different cell types [90, 91]. The CM exerted anti-inflammatory and antiapoptotic actions and induced the expression of BDNF and neurotrophin-3 (NT-3) in NSC-34 motor neurons exposed to scratch injury [90]. In particular, the CM revealed to contain NGF, NT3, IL-10, and TGF-*β* which may have a role in mediating the neuroprotective effect [90]. In addition, MSC CM was reported to attenuate staurosporine- (STS-) induced apoptosis in a concentration-dependent manner in both SOD1^G93A^ and nontransgenic primary motor neurons, NSC-34 cells, and astrocytes. This result may be explained by the fact that CM induced the expression of neurotrophic and growth factors and in parallel reduced the expression of the cytokines in both transgenic and nontransgenic astrocytes [91].

Another study investigated the effects of exosomes derived from ADMSCs on differentiated neuronal stem cells (NSCs) isolated from SOD1^G93A^ ALS mice. Results evidenced that exosomes were able to reduce SOD1 aggregation and prevented the abnormal expression of mitochondrial proteins and indeed normalized the phospho-CREB/CREB ratio and PGC-1a expression [92]. The exosomes derived from ADMSCs were also able to protect NSC-34 cells expressing ALS mutations from oxidative damage [93].

Interestingly, the infusion of a different type of cells, namely, Tregs, seems to be a new therapeutic option. Indeed, it was demonstrated that Tregs are able to suppress immune toxicity by inhibiting microglial activation, the proliferation of cytotoxic T lymphocytes providing motor neuron protection in ALS [94]. A recent phase I trial demonstrated the safety and potential benefit of autologous Treg infusions in ALS patients. Indeed, Treg administration slowed the progression rates during the early and later stages of the disease [95].

## 7. Conclusions

MSCs had already shown a therapeutic potential in different clinical fields. In particular, they may exert their action differentiating toward a specific cell type or through the releasing of different growth and trophic factors. ALS rodent models evidenced that MSCs may represent a promising approach to treat ALS; indeed, MSC transplantation delayed the disease onset and progression and increased the lifespan of treated animals. Furthermore, also the loss of motor neurons was reduced, showing a delay in motor function loss in MSC-treated animals. The results obtained from preclinical studies encouraged the MSC administration in ALS patients. The results in humans, although confirming the safety of MSC transplantation, showed only a partial improvement of ALS clinical scores and only some of them evidenced an effect in slowing down disease progression. For this reason, even if the first results may indicate a possible beneficial action of MSC transplantation in ALS, more studies and trials with a higher number of patients are necessary to obtain more significant results and to better understand the potential MSC mechanism of action.

## Figures and Tables

**Table 1:** Overview of the studies involving the administration of MSCs in in vivo rodent models of ALS.

MSC source	MSC administration	Number of cells	Rodent model	Delayed ALS onset/progression	Increased survival	Delayed the loss of motor function	Ref.
Human BMMSCs	Intravenous	3 × 10^6^ cells	Presymptomatic SOD1^G93A^ mice	Yes	Yes	Yes	[43]
Human BMMSCs	Intraspinal	1 × 10^5^ cells	Asymptomatic SOD1^G93A^ mice	Yes	Yes	Yes	[44]
Murine BMMSCs	Intravenous	1 × 10^6^ cells	Symptomatic SOD1^G93A^ mice	Yes	Yes	Yes	[45]
Rat BMMSCs	Intrathecal	2 × 10^6^ cells	Symptomatic SOD1^G93A^ rats	Yes	Yes	Yes	[46]
Human BMMSCs	Intracisternal	3 × 10^5^ cells	Early symptomatic SOD1^G93A^ mice	—	—	Yes	[47]
Human BMMSCs	Intrathecal	5 × 10^5^ cells at the age of 8, 10, and 12 weeks	SOD1^G93A^ mice	Yes	Yes	Yes	[49]
Rat BMMSCs	Intraspinal and intravenous	10^5^ cells (intraspinal); 2 × 10^6^ cells (intravenous)	SOD1^G93A^ rats	Yes	Yes	Yes	[50]
BMMSCs from an ALS patient	Intrathecal	1 × 10^4^, 2 × 10^5^, and 1 × 10^6^ cells	SOD1^G93A^ mice	No	Yes	Yes	[51]
Human BMMSCs	Intrathecal	5 × 10^5^ cells	SOD1^G93A^ rats	Yes	Yes	Yes	[52]
Murine ADMSCs	Intravenous	2 × 10^6^ cells	SOD1^G93A^ mice	Yes	No	Yes	[54]
Human ADMSCs	Intravenous or intracerebroventricular	1 × 10^6^ cells (intravenous); 2 × 10^5^ cells (intracerebroventricular)	SOD1^G93A^ mice	Yes	Yes	Yes	[55]
Human UC-MSCs	Intracerebroventricular or intravenous	2.5 × 10^5^ cells (lateral ventricles); 1 × 10^6^ cells (intravenous)	Early symptomatic SOD1^G93A^ mice	—	—	—	[56]
Human UC-MSCs	Intracerebroventricular	2.5 × 10^5^ cells at 14, 16, and 18 weeks. For behavioural test, animals received another injection at 20 weeks	SOD1^G93A^ mice	No	No	No	[57]
Human AMSCs	Intravenous	1 × 10^6^ cells at 12, 14, and 16 weeks	SOD1^G93A^ mice	Yes	Yes	Yes	[58]
Human BMMSCs expressing GDNF	Intramuscular	1.2 × 10^5^ cells twice with a one-week interval	SOD1^G93A^ rats	Yes	Yes	Yes	[59]
Human BMMSCs neurally induced by transduction with a retroviral vector encoding Ngn1	Intravenous	1 × 10^6^ cells before the onset of symptoms (8 weeks) or after (14–16 weeks).	SOD1^G93A^ mice	Yes	Yes	Yes	[61]

ALS: amyotrophic lateral sclerosis; AMSCs: amniotic mesenchymal stem cells; ADMSCs: adipose derived mesenchymal stem cells; BMMSCs: bone marrow derived mesenchymal stem cells; CNS: central nervous system; MSCs: mesenchymal stem cells; UC-MSCs: umbilical cord mesenchymal stem cells.

**Table 2:** Overview of the studies and clinical trials with available and published results involving the administration of MSCs in ALS patients.

NCT number/ethical approval	Clinical trial title	Phase	MSC source	MSC administration	Number of cells	Number of patients	Location	Major adverse events	Delayed disease progression	Ref.
NCT01759797	Intravenous Transplantation of Mesenchymal Stem Cell in Patients With ALS	Phase 1	Autologous BMMSCs	Intravenous	2 × 10^6^ cells/kg	6	Iran	No	No	[70]
NCT01771640	Intrathecal Transplantation of Mesenchymal Stem Cell in Patients With ALS	Phase 1	Autologous BMMSCs	Intrathecal	2 × 10^6^ cells/kg	8	Iran	No	No	[70]
NCT01363401	Safety and Efficacy Study of Autologous Bone Marrow Derived Stem Cell Treatment in Amyotrophic Lateral Sclerosis	Phase 1/2	Autologous BMMSCs	Two repeated intrathecal injections	1 × 10^6^ cells/kg, range 48-86 × 10^6^ cells	8: phase 1; 64: phase 2	Korea	No	Yes	[71, 73]
NCT01609283	A Dose-escalation Safety Trial for Intrathecal Autologous Mesenchymal Stem Cell Therapy in Amyotrophic Lateral Sclerosis	Phase 1	Autologous ADMSCs	Intrathecal	Group 1: 1 × 10^7^ cells; Group 2: 5 × 10^7^ cells; Group 3: 5 × 10^7^ cells (2 doses); Group 4: 1 × 10^8^ cells; Group 5: 1 × 10^8^ cells (2 doses)	27	United States	No	No	[74]
NCT01777646	Autologous Mesenchymal Bone Marrow Stromal Cells Secreting Neurotrophic Factors (MSC-NTF), in Patients With Amyotrophic Lateral Sclerosis (ALS)	Phase 2	BMMSCs induced to secrete neurotrophic factors	Both intramuscular and intrathecal	Low dose: 1 × 10^6^ cells/kg intrathecal and 24 × 10^6^ cells intramuscular; mid-dose: 1.5 × 10^6^ cells/kg intrathecal and 36 × 10^6^ cells intramuscular; and high dose: 2 × 10^6^ cells/kg intrathecal and 48 × 10^6^ cells intramuscular	14	Israel	No	Yes	[75]
NCT01051882	Autologous Cultured Mesenchymal Bone Marrow Stromal Cells Secreting Neurotrophic Factors (MSCNTF), in ALS Patients.	Phase 1/2	BMMSCs induced to secrete neurotrophic factors	Intramuscular injections at 24 separate sites or intrathecal administration.	1 × 10^6^ cells/site (intramuscular); 1 × 10^6^/kg cells (intrathecal)	12	Israel	No	Yes	[75]
NCT02881476	Therapeutic Treatment of Amyotrophic Lateral Sclerosis	Phase 1	Allogeneic Wharton's jelly-derived MSCs	Intrathecal	Average dose of 0.42 × 10^6^ cells/kg	43	Poland	No	—	[76]
NCT02881489	Autologous Bone Marrow Mesenchymal Stem Cells in the Treatment of Patients With Amyotrophic Lateral Sclerosis (UwmBmmscALS)	Phase 1	Autologous BM-MSCs	Intrathecal	Mean: 15 × 10^6^	30	Poland	No	Yes/no	[77]
Ethical Committee of the Piedmont Region	—	—	Autologous BMMSCs	Intraspinal	—	7	Italy	No	—	[78]
Ethical Committee of the Piedmont Region	—	—	Autologous BMMSCs	Intraspinal	Range: 7.0–152 × 10^6^	9	Italy	No	Yes	[79]
Ethical Committee of the Piedmont Region	—	—	Autologous BMMSCs	Intraspinal	Range: 7.0–152 × 10^6^	9	Italy	No	Yes	[80]
Registration number 16454-pre21-823; approved and monitored by the National Institute of Health and by the Ethics Committees of the Piedmont Region, the “Maggiore della Carità” and “San Giovanni Bosco” Hospitals	—	Phase 1	Autologous BMMSCs	Intraspinal	Range: 11.4-120 × 10^6^ cells	10	Italy	No	—	[81]
Registration numbers 12947-29.3 and 16454-pre21-823; approved and monitored by the ethics committees of the Piedmont Region, ‘Maggiore della Carità' and San Giovanni Bosco hospitals and the National Institute of Health, Rome	—	Phase 1	Autologous BMMSCs	Intraspinal	Range: 7.0 × 10^6^–152 × 10^6^	19	Italy	No	No	[82]
Ethics committees of the Gennimatas General Hospital and Hadassah Hebrew University Hospital and registered in the National Institutes of Health database	—	Phase 1/2	Autologous BMMSCs	Intrathecal. 9 patients also received intravenous MSCs	Mean 54.7 × 10^6^ cells. 9 patients also received intravenous MSCs: mean 23.4 × 10^6^	19	Israel	No	—	[83]
IRB (HYUH IRB 2006-339) and the Korean Food and Drug Association (KFDA, Bio-47)	—	—	Autologous BMMSCs	Intrathecal (two monthly doses)	1 × 10^6^ cells per kg	37	South Korea	—	Yes/no	[84]
EudraCT No. 2011-000362-35; State Institute for Drug Control and by the ethics committee of the University Hospital Motol in Prague, Czech Republic	—	Phase 1/2a	Autologous BMMSCs	Intrathecal	15 ± 4.5 × 10^6^ cells	26	Czech Republic	No	Yes	[85]
This study was approved and supported by Shefa Neuroscience Research Center, Tehran, Iran (N.S.R.C.sh89-1).	—		Bone marrow-derived neural stem cells	Intraspinal	5 × 10^6^ cells	8	Iran	No	Yes	[86]
Ethics Committee of Republican Research and Practical Center of Neurology and Neurosurgery	—	—	Autologous BMMSCs committed to neuronal differentiation	Intravenous. Neural induced MSC were injected via lumbar puncture	Untreated cells: 0.5-1.5 × 10^6^/kg body weight; range: 42-102 × 10^6^ cells. Neural induced MSCs: 5.0-9.7 × 10^6^ cells	10	Belarus	No	Yes	[87]

ADMSCs: adipose derived mesenchymal stem cells; ALS: amyotrophic lateral sclerosis; BMMSCs: bone marrow mesenchymal stem cells; MSCs: mesenchymal stem cells; UC-MSCs: umbilical cord mesenchymal stem cells.
